# Self-assessment of dental health status, behaviours and oral health risk factors among adolescents from public schools in Maputo City-Mozambique

**DOI:** 10.1186/s12903-023-03742-0

**Published:** 2024-01-31

**Authors:** Amália Issufo Mepatia, Neil Myburgh, Robert Barrie, Faheema Kimmie-Dhansay

**Affiliations:** Department of Community Oral Health, Faculty of Dentistry, University of the Western Cape, Maputo, South Africa

**Keywords:** Oral health, Oral Health risk factors, Oral health behaviour, Oral health self-assessment, Dental visits, Adolescents, Urban and peri-urban schools

## Abstract

Self-assessment of dental health status may have an impact on the oral health behaviour of adolescents which could impact their oral health. Oral health has been linked to various medical health conditions, thus eliminating oral health diseases can improve general health. The present study aimed to assess the association between behaviours and risk factors (oral hygiene habits, sugar intake, urban/rural status) and negative self-perception of dental health status among adolescents attending public schools in Maputo City.

**Method** An analytic cross-sectional study, conducted in three Primary public schools from urban and peri-urban areas in Maputo City selected by convenience due to their geographic location was included. The size of the sample was 236 12-year-olds. Data was collected using a self-completion questionnaire designed by the World Health Organization (WHO). Chi-square tests or Fishers’ Exact tests were used for associations. A simple and multiple logistic regression was used to determine the strength of these associations using backward elimination (*p* < 0.05). Results: The sample consisted of 221 adolescents, with 114 (51.6%) residing in urban areas and 107 (48.4%) in peri-urban areas. More than half of the participants (111 individuals) reported having a negative perception of their dental health. In the urban location, a higher percentage of participants had a “negative” perception of dental health (57.9%, *n* = 66), while in the peri-urban location, more participants perceived their dental health as “positive” (57.9%, *n* = 62). Participants residing in an urban setting were 82% more likely to have a negative perception of dental health (AOR = 1.82 [95% C.I.: 1.05 to 3.14]). Those who had experienced dental pain tended to report a higher proportion of negative dental perception (57.2%, *n* = 91), with 2.7 times more likely to report a negative perception of dental health (AOR = 2.72 [95% C.I.: 1.46 to 5.08]). The majority (*n* = 139; 63.2%) claimed to clean their teeth twice a day.

**Conclusion** There was a higher negative perception of dental health in urban areas. The need to strengthen oral health promotion in urban schools is high since schools play such a significant role in oral health promotion.

## Introduction

According to the World Health Organization, “*Oral Health is a state of being free from mouth and facial pain, oral and throat cancer, oral infection and sores, periodontal (gum) disease, tooth decay, tooth loss, and other diseases and disorders that limit an individual capacity in biting, chewing, smiling, speaking, and psychosocial wellbeing*” [1]. The exposure to pain, limitation with using the teeth, smiling and communication due to missing and poor appearance of teeth, have a considerable impact on people´s daily lives and well-being, resulting in reduced vivacity at school and at home, and causing the loss of millions of hours of work each year all over the world [2].

Oral diseases are qualified as a big health problem due to its prevalence and incidence in the different regions. The treatment cost is high in the industrialized countries and almost not feasible in most developing countries [3]. Some studies have demonstrated that a population´s perceptions and beliefs about disease may strongly impact their behaviours [4]. Literature shows that studying oral health perceptions could contribute with important knowledge that can lead to improvement in public oral health and proper use of health care services [5].

During adolescence, lifestyle behaviours such as eating and hygiene, physical activity, tobacco consumption, alcoholic beverages, or drugs, which may influence the morbidity pattern in adulthood habits, are being formed. However, it is a period in which increased learning related to positive attitudes and behaviours that, built in a decisive way, are important to promote oral health [6]. Therefore, it is important to study adolescents from their perspective and vision of the world to develop more effective measures, and plan actions for this specific target group [7].

Studies about oral health in Mozambique are scarce in the literature, which does not allow us to have the real panorama of oral health status in the country [8]. The results of the dental survey for Mozambique highlights the dental disease burden, but it does not include dental risk factors or the self-perception of oral health [9]. There is no data on the access to dental services in the urban and peri-urban areas around Maputo City in Mozambique. There is also no data on the oral health risk factors facing adolescents in the urban and peri-urban areas of Mozambique. Information on the risk factors such as toothbrushing frequency, use of toothpaste, healthy eating habits; self-assessment of oral health status; and oral health behaviours can be helpful when designing oral health promotion strategies aimed at adolescents in Mozambique. The aim of the present study was to assess the association of sex, school location, experience of dental pain, use of dental service, reasons for last dental visit, oral health behaviour, toothbrushing behaviour, use of fluoridated toothpaste, and sugar intake and negative self-perception of dental health status among adolescents attending public schools in Maputo City.

## Methods

A cross-sectional study was carried out from May to June 2018 in three public schools in urban and peri-urban areas in Maputo city. The study was reported according to the STROBE guidelines [10]. Study site selection was by convenience, due to greater operational ease and low cost. Two primary schools from each area were selected. The list of students who were 12-years old was obtained through the teacher responsible for each class. To obtain a representative number to maximize sample size, 95% of confidence level was set with a margin for error of 5%. The sample size calculation was based on a national survey results from Mozambique conducted in seven provinces where the dental caries prevalence was 19.1% for adolescents aged 12 years old [11].

A structured questionnaire designed by World Health Organization for children was used to collect data. The original questionnaire was in English and translated into Portuguese, which was first used for a pilot study in April 2018 involving 20 students of 12 years old from schools of both areas, not covered by main study. The questionnaire was not back translated, and the validity of the new questionnaire was not performed. The percentage of answers from these students in the pilot study was more than 90%.

The outcome of interest (dependent variable) was the self-assessment of dental health was scored from, “very bad” to “excellent”. Self-assessment of dental health was then dichotomized where “very bad”, “bad” and “normal” was classified as a “negative perception of dental health” and “good”, “very good” and “excellent” was classified as a “positive perception of dental health”. Dental services utilization (dental visits) was also dichotomized into “never visited a dentist” and “visited the dentist at least once”.

The independent variables included socio-demographic factors, experience of dental pain or discomfort related to teeth, use of dental service, oral health behaviour and dietary intake. The sociodemographic factors were sex and school location (urban and peri-urban). The use of dental services was measured by participants’ dental visits within the past 12 months and the reason for their last visit, categorized as “Pain,” “Treatment/Follow up/Check-up,” or “Missing.” Additionally, oral health behaviours were considered, including toothbrushing frequency (< once a day or > = twice a day), use of fluoridated toothpaste, and frequency of consuming certain foods like fruits, biscuits/cake, and sugar drinks (< once a day or > = twice a day, even in small quantities”). Participants who added sugars to their milk, tea, or coffee, or consumed sugar-sweetened beverages were classified as having access to “drinks with sugar.”

Chi-square tests or Fishers’ Exact tests were used for associations. A simple and multiple logistic regression models were used to determine the strength of these associations using backward elimination. All tests were assumed statistically significant at *p* < 0.05. All tests were performed using StataCorp. 2021. Stata Statistical Software: Release 17. College Station, TX: StataCorp LLC.

## Results

The sample comprised 221 adolescents aged 12 years old’s attending public schools in the urban (*n* = 114; 51.6%) and peri-urban area (*n* = 107; 48.2%) of Maputo City. Over 50% (*n* = 111) felt that they had a negative perception of dental health and 49.8% (*n* = 110) felt that they had a positive perception of their own dental health. There were more females (*n* = 126; 57.0%) compared to males (*n* = 95; 43.0%) participants. Just over 70% (71.0%, *n* = 159) reported to have experienced dental pain. 

Most of the sample reported to have visited the dentist at least once (67.9%, *n* = 150). The majority of the sample (63.2%, *n* = 139) reported brushing their teeth twice daily and using fluoridated toothpaste (Table 1).

**Table 1:** Association of sociodemographic variables, behaviours, and risk factors (oral hygiene habits and sugar intake) with self-assessed dental health in 12-year-old children in Mozambique

Healthy teeth perceptions in 12-year-olds			Self-assessed dental health perceptions n (%)		
		Total n (%)	Positive dental perception 110 (49.8)	Negative dental perception 111 (50.2)	*p*-value
Sociodemographic variables					
Sex	Female	126 (57.0)	61 (48.4)	65 (51.6)	0.641
	Male	95 (43.0)	49 (51.6)	46 (48.4)	
Location	Urban	114 (51.6)	48 (42.1)	66 (57.9)	0.019*
	Peri-Urban	107 (48.2)	62 (57.9)	45 (42.1)	
Dental pain or discomfort related to teeth,	Never	62 (28.1)	42 (67.7)	20 (32.3)	0.001*
	Sometimes	159 (71.9)	68 (42.8)	91 (57.2)	
Use of dental services					
Dental Visits within the past 12 months	Never ever	71 (32.1)	36 (50.7)	35 (49.3)	0.849
	At least once	150 (67.9)	74 (49.3)	76 (50.7)	
Reasons for last dental visits	Pain	50 (31.2)	24 (48.)	26 (52.)	0.817
	Treatment/Follow up/Check up	100 (45.3)	50 (50.)	50 (50.)	
	Missing	71 (32.1)			
Oral health behaviours and dietary intake					
Toothbrushing frequency	< once a day	81 (36.8)	40 (49.4)	41 (50.6)	0.889
	>=twice a day	139 (63.2)	70 (50.4)	69 (49.6)	
Fluoridated toothpaste usage	No	16 (7.3)	8 (50.)	8 (50.)	0.985
	Yes	203 (92.7)	101 (49.8)	102 (50.3)	
Eat fruit	Never	148 (75.9)	74 (50.)	74 (50.)	0.899
	At least once a day	47 (24.1)	23 (48.9)	24 (51.1)	
Eat biscuits and cake	Never	165 (91.7)	82 (49.7)	83 (50.3)	0.472
	At least once a day	15 (8.3)	6 (40.)	9 (60.)	
Drinks with sugar	Never	143 (64.7)	71 (49.7)	72 (50.4)	0.96
	At least once a day	78 (35.3)	39 (50.)	39 (50.)	

There was a statistically significant association between of school location (urban/peri-urban) and dental pain with self-assessment of dental health. There was no association between sex, use of dental service, oral health behaviours and sugar intake and self-assessed dental perception.

An unadjusted odds ratio was run and determined that participants from an urban location were more likely to report a negative perception of dental health (UOR = 1.89 [95% C.I: 1.11 to 3.23]) compared to participants from a peri-urban location. It was also determined that participants with dental pain were more likely to report a negative perception of dental health (UOR = 2.81 [95% C.I.: 1.51 to 5.22]).When a multiple logistic regression model was run, both the AOR for toothache and rural school location remained the similar (Table 2).

**Table 2:** Unadjusted and adjusted logistic regression models for Negative self-perception of oral health

				UOR (95% C.I)		*p*-value		AOR		*p*-value
Sex (Female)		Male		0.88 (0.52 to 1.5))		0.641				
School location (Peri-Urban)		Urban		1.89 (1.11 to 3.23))		0.019*		1.82 (1.05 to 3.14)		0.033*
Toothache (Never)		Sometimes		2.81 (1.51 to 5.22))		0.001*		2.72 (1.46 to 5.08)		0.002*
Reason for dental visit (Pain)		Check-up		0.92 (0.47 to 1.82))		0.817				
Frequency of cleaning teeth (< once a day)		>=twice a day		0.96 (0.56 to 1.66))		0.889				
Use of fluoridated toothpaste (No)		Yes		1.01 (0.36 to 2.79))		0.985				
Eat Fruit (Never)		Eat fruit (at least once a day)		1.04 (0.54 to 2.01)		0.899				
Eat Biscuit (Never)		Eat biscuit (at least once a day)		1.48 (0.5 to 4.35))		0.474				
Drinks with sugar (Never)		Drinks (at least once a day)		0.99 (0.57 to 1.71))		0.96				

*UOR* Unadjusted Odds ratio, *AOR *Adjusted Odds Ratio, *95%C.I. *95% Confidence Interval); * Statistically significant

## Discussion

The aim of this study was to determine the behaviour and risk factors and negative self-perception of dental health status of adolescents in Maputo. Just over 50% of the sample felt that they had a negative perception of their dental health. In this study, dental pain (toothache) was associated with negative self-assessed oral health. This finding corroborates the findings in elders by Pattussi et al. [12].

There was no difference in self-assessed dental health between males and females in this study. Males were more likely to report poor self-assessed oral health in elders in Pattussi et al. [12] which differed from this study. In a household study conducted in South Africa by Olutula et al. [13], it was determined that males rated their oral health as “good” compared to females. Pengpid and Peltzer, in a household study in Sudan, reported that females had a higher odds of poor self-reported oral health [14], which differed from the findings in this study. The differences in the above studies could be attributed to the differing ages groups across the various studies.

In this paper, it was found that there was higher frequency of negative self-assessed dental health among those adolescents living in peri-urban areas. This could be attributed to lack of access to dental care in the peri-urban areas. In addition, participants from urban schools were more likely to report an increased odds of negative self-perceived dental health compared to their peri-urban counterparts. However, Pattussi et al. [12] found that elders experienced no difference in the self-rated oral health between participants living in rural or urban areas. 

Silva et al. [15] states that the untreated carious teeth were related to poor self-assessed dental health. In this study, dental pain was associated with negative self-assessed dental health. A higher proportion of participants who reported to, “sometimes have pain”, had a higher negative self-reported dental assessment, and reported almost three times the odds of experiencing a negative self-reported dental health when experiencing dental pain sometimes compared to never experiencing dental pain. Varenne et al. [15] reported a lower dental pain compared to this study, while only 8% of the study participants reported “poor” oral health in Burkina Faso.

The increased intake of cariogenic food and drinks, as well as inadequate oral hygiene techniques are probably the main causes of heightened dental caries activity in adolescence. Therefore, the use of fluoridated toothpaste as a topical fluoride on the tooth surface and dental floss to remove plaque is crucial. Personal education and motivation about oral health based on oral hygiene needs and disease patterns are highly recommended, as well as the professional follow up for this group in particular [16]. The results in the study conducted by [8] in Maputo City in Mozambique involving adolescents aged 12 years, reported a low consumption of sugar. The present study showed a higher prevalence of sugar intake, in which 35.3% drink tea, coffee or milk with added sugar every day, and more than 8% consume biscuits and cakes. Kahabuka et al. [17] reported that 61.6% of their study’s population reported to never eat biscuits and sweets, while 57.9% reported to eat fruit at least once a day.

A study conducted in Maputo Province evaluated oral hygiene habits of patients attending three health centers in Maputo Province who volunteered to be part of a study. The majority (99.17%) reported the use of conventional toothbrush and toothpaste. 80% of the participants said they brush their teeth twice a day, while 10.83% said once a day and only 9.17% brush three times a day [18]. This was much higher than in our study. In addition, Kahabuka et al. [17] reported that 92% of their study population brushed their teeth twice a day, which is also much higher than this current study.

Various national challenges exists as pointed out in Kwan et al. [19, 20] which alluded to the cost of running and implementing health promotion programmes. Income from tuck shops or food vendors, which the schools benefit from, has an impact on the success of oral health promotion programmes, as they provide cheap sugary food and drinks to the school learners which are detrimental to the overall health and wellbeing of the learners. Garnering support from parents and teachers alike will sustain any oral or health promotion programme. However, an over-saturated curriculum will hinder teachers from adding an additional oral health promotion programme into their already comprehensive curriculum [19]. Implementing oral health promotion programmes may have more challenges when implementing it in lower-income countries due to various challenges such as hunger, gender disparity, poverty, and political unpredictability [20, 21].

In the current study over 32.1% of the participants never visited a dentist. In the Brazilian survey, results showed that only 18% of adolescents aged 12 years had never been to a dentist, whilst our study reported a prevalence of 32.1% [22]. A study conducted in Maputo Province showed that most of the participants (77%) never visited a dentist, and of those who did, 40% visited at least once a year. As for the reasons, 61.5% visited a dentist for an extraction, 30.9% because of pain and only 4.8% go for a routine check-up [23]. In a study in Burkina Faso, 100% of 12-year-old children living in urban areas reported to visit a dentist compared to children living in a rural area [15]. In Cameroon, over 90% of the study sample never visited a dentist, irrespective of their urban or rural location [24].

According to Oral Health Annual Report, 52% of the treatment in the dental services is tooth extraction and only 9.7% is dental fillings at national level. In Maputo City, these tooth extractions correspond to 17.6% and the dental fillings are 23.8% of the total treatments in the country [11].

## Conclusion and recommendations

Dental pain and living in urban setting were significantly associated with higher frequency of negative self-perceived oral health. Negative self-perceived oral health has an impact on oral health measures and thus essential when planning strategies to promote good oral health.

### Limitations

Clinical variables were not included (such as caries disease), a non-validated WHO questionnaire was used. The questionnaire was not back translated, and the validity of the new questionnaire was not performed. A non-probabilistic sample was selected. All these factors have an impact on the external validity of the results and the results should be read with caution.

## Data Availability

The datasets used and/or analysed during the current study available from the corresponding author on reasonable request.
